# Psychotic-Like Experiences and Nonsuidical Self-Injury in England: Results from a National Survey

**DOI:** 10.1371/journal.pone.0145533

**Published:** 2015-12-23

**Authors:** Ai Koyanagi, Andrew Stickley, Josep Maria Haro

**Affiliations:** 1 Parc Sanitari Sant Joan de Déu, Universitat de Barcelona, Fundació Sant Joan de Déu, Sant Boi de Llobregat, Barcelona, Spain; 2 Instituto de Salud Carlos III, Centro de Investigación Biomédica en Red de Salud Mental, (CIBERSAM), Madrid, Spain; 3 The Stockholm Centre for Health and Social Change (SCOHOST), Södertörn University, Huddinge, Sweden; 4 Department of Human Ecology, Graduate School of Medicine, The University of Tokyo, Tokyo, Japan; Katholieke Universiteit Leuven, BELGIUM

## Abstract

**Background:**

Little is known about the association between psychotic-like experiences (PLEs) and nonsuicidal self-injury (NSSI) in the general adult population. Thus, the aim of this study was to examine the association using nationally-representative data from England.

**Methods:**

Data from the 2007 Adult Psychiatric Morbidity Survey was analyzed. The sample consisted of 7403 adults aged ≥16 years. Five forms of PLEs (mania/hypomania, thought control, paranoia, strange experience, auditory hallucination) were assessed with the Psychosis Screening Questionnaire. The association between PLEs and NSSI was assessed by multivariable logistic regression. Hierarchical models were constructed to evaluate the influence of alcohol and drug dependence, common mental disorders, and borderline personality disorder symptoms on this association.

**Results:**

The prevalence of NSSI was 4.7% (female 5.2% and male 4.2%), while the figures among those with and without any PLEs were 19.2% and 3.9% respectively. In a regression model adjusted for sociodemographic factors and stressful life events, most types of PLE were significantly associated with NSSI: paranoia (OR 3.57; 95%CI 1.96–6.52), thought control (OR 2.45; 95%CI 1.05–5.74), strange experience (OR 3.13; 95%CI 1.99–4.93), auditory hallucination (OR 4.03; 95%CI 1.56–10.42), and any PLE (OR 2.78; 95%CI 1.88–4.11). The inclusion of borderline personality disorder symptoms in the models had a strong influence on the association between PLEs and NSSI as evidenced by a large attenuation in the ORs for PLEs, with only paranoia continuing to be significantly associated with NSSI. Substance dependence and common mental disorders had little influence on the association between PLEs and NSSI.

**Conclusions:**

Borderline personality disorder symptoms may be an important factor in the link between PLEs and NSSI. Future studies on PLEs and NSSI should take these symptoms into account.

## Introduction

Nonsuicidal self-injury (NSSI) refers to the deliberate, self-inflicted damage of bodily tissue without the intention to die [1]. Previously reported lifetime prevalence of NSSI has ranged from 4–45% and 19–60% in community-based and clinical samples respectively [2,3]. Common methods of NSSI include cutting, burning, scratching, and self-hitting, with the level of resulting injuries ranging from superficial wounds to severe mutilation. Although some overlapping features exist between self-injury and intention to die, there is growing recognition that NSSI and suicidal behavior are distinct entities as evidenced by significant differences in terms of etiology, psychiatric impairment, psychological function, method of self-harm, and course or outcome between the two phenomena [4].

Although the age of onset of NSSI is commonly in adolescence, NSSI is known to occur across the entire lifespan [5]. NSSI is associated with adverse health outcomes such as severe injury including nerve damage, risk of infection, accidental death, and future suicide attempts [4], while it also carries significant societal costs [6]. The typical motivation for NSSI is to relieve negative emotions or to solve interpersonal difficulties [7]. Prior versions of the Diagnostic and Statistical Manual (DSM) (e.g. DSM-IV) included self-harming behavior only in one of the nine diagnostic criteria for borderline personality disorder. However, research has shown that this behavior is observed in a spectrum of other psychiatric disorders such as anxiety, depression, PTSD, eating disorders, and substance abuse, and that it often occurs in individuals without borderline personality disorder [4]. These findings have led to the inclusion of NSSI as a condition for further study in DSM-5 [8].

Despite growing interest in NSSI research in recent years [1], one mental health condition that has been little studied in relation to NSSI is subclinical psychosis or psychotic-like experiences (PLEs). PLEs are highly prevalent in the general population, with figures of 20% or above being reported in some studies [9,10]. They can be defined as the manifestation of psychotic symptoms such as delusions or hallucinations that do not reach the clinical threshold in terms of the level of associated distress and treatment need [11]. The only study to date on PLEs and NSSI is a recent school-based study among Australian adolescents which also focused on the effects of psychological distress in relation to NSSI [12]. This study found that PLE alone (without psychological distress) was associated with NSSI cross-sectionally but not longitudinally. However, persistence of PLEs predicted the future occurrence of NSSI. The number of variables used for adjustment in this study was limited and did not include potentially important factors such as stressful life events, common mental disorders (CMDs) and borderline personality disorder symptoms. Furthermore, this study did not assess individual PLE symptoms despite the dimensional differences that may exist between the distinct types of psychotic symptoms [13–15]. Thus, the aim of the current study was to assess the association between different types of PLE and NSSI using a nationally-representative dataset of the English adult population aged 16–95 years. In particular, we focused on the influence of borderline personality disorder symptoms, CMDs, and alcohol and drug dependence on the association between PLEs and NSSI not only for their clinical relevance but also because these factors have never been assessed in previous studies despite the fact that they may be strong confounders in the association between PLEs and NSSI [2,4,16–20]. As a majority of people with PLEs and/or NSSI do not seek medical care [8,21], community-based studies are most appropriate for a comprehensive understanding of the association between PLEs and NSSI.

## Methods

### The survey

We analyzed data from 7403 people who provided information for the Adult Psychiatric Morbidity Survey 2007 (APMS). The dataset (catalogue number SN6379) is publically available though the UK data archive (http://www.data-archive.ac.uk/) and any interested researcher may apply for this dataset. Details of the survey have been published previously elsewhere [22,23]. This was a representative survey of the adult population in England (aged 16 years and above) residing in private households. Fieldwork was undertaken between October 2006 and December 2007 by the National Center for Social Research, together with Leicester University. A multistage stratified probability sampling design was used, with the user Postcode Address File (PAF) serving as the sampling frame and individual or groups of postcode sectors as the primary sampling units (PSUs). Postcode sectors were stratified by Strategic Health Authority (SHAs) regions and by socio-economic groupings and household (non-)car ownership, with 519 postal sectors finally being selected. Within each of these, 28 delivery points were randomly chosen. This resulted in a total sample of 14,532 delivery points. After exclusions of non-residential addresses or addresses of unknown eligibility, 13,171 addresses were either eligible or probably eligible for participation. When there were two or more people in a household, one was randomly selected. Information on the participant was obtained by both face-to-face computer-assisted personal interviewing (CAPI) and computer-assisted self-completion interviews (CASI). CASI was conducted for particularly sensitive questions by handing the laptop to the participant for him or her to enter the answers to the questions. The respondent was informed beforehand that the interviewer cannot access the results of the self-completed information. From the 13,171 eligible households, 7,461 adults participated, giving a survey response rate of 57%. To ensure that the sample was representative of the English adult population (i.e. to adjust for survey non-response and the probability of being selected), sampling weights were generated. Ethical approval for the study was given by The Royal Free Hospital and Medical School Research Ethics Committee (reference number 06/Q0501/71). Written consent was obtained from all participants.

### Variables

#### Nonsuicidal self-injury (NSSI)

NSSI was assessed by the question "Have you ever deliberately harmed yourself in any way but not with the intention of killing yourself?" Those who answered affirmatively were considered to have engaged in NSSI behavior [24]. In the APMS, this question was asked in two ways (i.e. during face-to-face and self-completion interviews). We used information obtained from the self-completion interview for the current analysis, as we believe this to be more accurate.

#### Psychotic symptoms

The presence of five types of psychotic symptom (mania/hypomania, thought control, paranoia, strange experience, and auditory hallucination) occurring in the past-12 months was assessed with the Psychosis Screening Questionnaire (PSQ) [25]. One or two follow-up questions after the main probe question for each type of psychotic symptom were used to determine the severity of the symptoms. We used the strictest criteria to define the presence of psychotic symptoms in an attempt to capture clinically relevant psychotic symptoms [26]. The questions and response options required for the endorsement of each PLE were as follows:

1. **Mania/Hypomania**: Over the past year, have there been times when you felt very happy indeed without a break for days on end? (yes)
  1a Was there an obvious reason for this? (no)
  1b Did people around you think it was strange? (yes)
2. **Thought control**: Over the past year, have you ever felt that your thoughts were directly interfered with or controlled by some outside force or person? (yes)
  2a Did it come about in a way that many people would find hard to believe, for instance, through telepathy? (yes)
3. **Paranoia**: Over the past year, have there been times when you felt that people were against you? (yes)
  3a Have there been times when you felt that people were deliberately acting to harm you or your interests? (yes)
  3b Have there been times when you felt that a group of people was plotting to cause you serious harm or injury? (yes)
4. **Strange experience**: Over the past year, have there been times when you felt that something strange was going on? (yes)
  4a Was it so strange that other people would find it very hard to believe? (yes)
5. **Auditory hallucination**: Over the past year, have there been times when you heard or saw things that other people could not? (yes)
  5a Did you at any time hear voices saying quite a few words or sentences when there was no one around that might account for it? (yes)

#### Sociodemographic variables

These consisted of age (16–34, 35–59, ≥60 years), sex, equivalized income tertiles (high ≥£29826, middle £14,057-<£29826, low <£14,057), education qualification (degree, non-degree, A-level, GCSE, other): yes or no), and ethnicity (white British or other).

#### Stressful life events

The stressful life events variable was the total number of 18 potentially stressful life events such as those pertaining to serious illness, death of an immediate family member, major financial crises, sexual abuse etc (S1 Table).

#### Alcohol dependence

Those with an Alcohol Use Disorders Identification (AUDIT) score ≥10 [27] were assessed for alcohol dependence with the community version of the Severity of Alcohol Dependence Questionnaire (SADQ-C) [28]. A score of four and above (out of 60) on the SADQ-C was used to determine alcohol dependence in the past 6-months.

#### Drug dependence

Those who reported that they had used at least one of eight types of drugs (cannabis, amphetamines, crack, cocaine, ecstasy, tranquilizers, opiates, or volatile substances) in the past 12 months were asked five questions based on the Diagnostic Interview Schedule [29] to determine the presence of past 12-month drug dependence.

#### Common mental disorder (CMD)

The Clinical Interview Schedule Revised (CIS-R) was used to determine the presence of six categories of CMD (depressive episode, mixed anxiety and depression, generalized anxiety disorder, panic disorder, phobia, and obsessive-compulsive disorder). The CIS-R identifies non-psychotic symptoms in the prior week to generate ICD-10 diagnoses, and can be administered by lay interviewers. Any CMD referred to the endorsement of at least one of the six CMDs [30].

#### Borderline personality disorder symptoms

The presence of the nine diagnostic criteria for borderline personality disorder was assessed by the Structured Clinical Interview for DSM-IV Axis II disorders [31]. The scores from each of the criteria (yes = 1 and no = 0) were added to create a scale ranging from 0–9 (Cronbach's alpha 0.74). As the inclusion of the item on suicide/self-injury may overinflate the association between borderline personality disorder symptoms and NSSI, while there may be colinearity between the item on paranoia/loss of contact with reality and some types of PLEs, we also created scales excluding either one or both of these criteria for the purpose of sensitivity analyses [20,32]. The scales were used as a continuous variable in the analysis, as subthreshold borderline personality disorder is also likely to be associated with NSSI [4].

#### Definite or probable psychosis

Individuals fulfilling at least one of the four phase-one psychosis screening criteria (current antipsychotic use, hospitalization for mental problems, endorsement of question 5a on auditory hallucination of the PSQ, and a self-reported diagnosis or symptoms of psychosis) were invited for a phase-two assessment. A definite diagnosis of psychosis (schizophrenia and affective psychosis) was based on the Schedules for Clinical Assessment in Neuropsychiatry (SCAN, version 2.1) [33] conducted in phase-two by a clinical interviewer. However, since 39% of those individuals invited for a phase-two interview either refused or could not be contacted, a ‘probable psychosis’ measure was also created for individuals without a phase-two interview but who fulfilled at least two of the phase-one psychosis screening criteria [23].

### Statistical analysis

The analyses were done with Stata version 13.1 (Stata Corp LP, College Station, Texas). As the focus of the study was on PLEs not reaching the clinical threshold for a psychosis diagnosis, we excluded those with definite or probable psychosis from the analysis. Multivariable logistic regression analysis was conducted with NSSI as the outcome. The main exposure variable of interest was PLEs. We assessed five different types of PLE (mania/hypomania, thought control, paranoia, strange experience, auditory hallucination) [26]. Any PLE referred to the endorsement of at least one of the five PLEs. Four different models were constructed: Model 1—adjusted for sex, age, education, ethnicity, income, and number of stressful life events; Model 2—in addition to the variables included in Model 1, alcohol and drug dependence, and CMDs were also adjusted for; Model 3—adjusted for the variables in Model 1 and borderline personality disorder symptoms; Model 4—adjusted for the variables in Model 1 and alcohol and drug dependence, CMDs and borderline personality disorder symptoms. This hierarchical analysis was undertaken in order to assess the effect of including substance dependence, CMDs, and borderline personality disorder symptoms in models adjusted for other confounders by comparing the odds ratios (ORs) of PLEs between the models. The selection of the control variables was based on past literature [19,20,34]. As nearly one-fifth of the participants had missing income information, a missing category was included to avoid omitting a large number of participants from the analysis. With the exception of stressful life events and borderline personality disorder symptoms (continuous variables), all variables were included in the models as categorical variables. In order to assess the influence of multicolinearity, we calculated the variance inflation factor (VIF) value for each independent variable. The highest VIF was 1.83, which is much lower than the commonly used-cut off of 10 [35], indicating that multicolinearity was unlikely to be a problem in our analyses. In order to generate nationally-representative estimates, in all analyses, the sample weighting and the complex study design were taken into account with Taylor linearization methods. ORs and 95% confidence intervals (95%CIs) are reported. The level of statistical significance was set at P<0.05.

## Results

**Fig 1:**
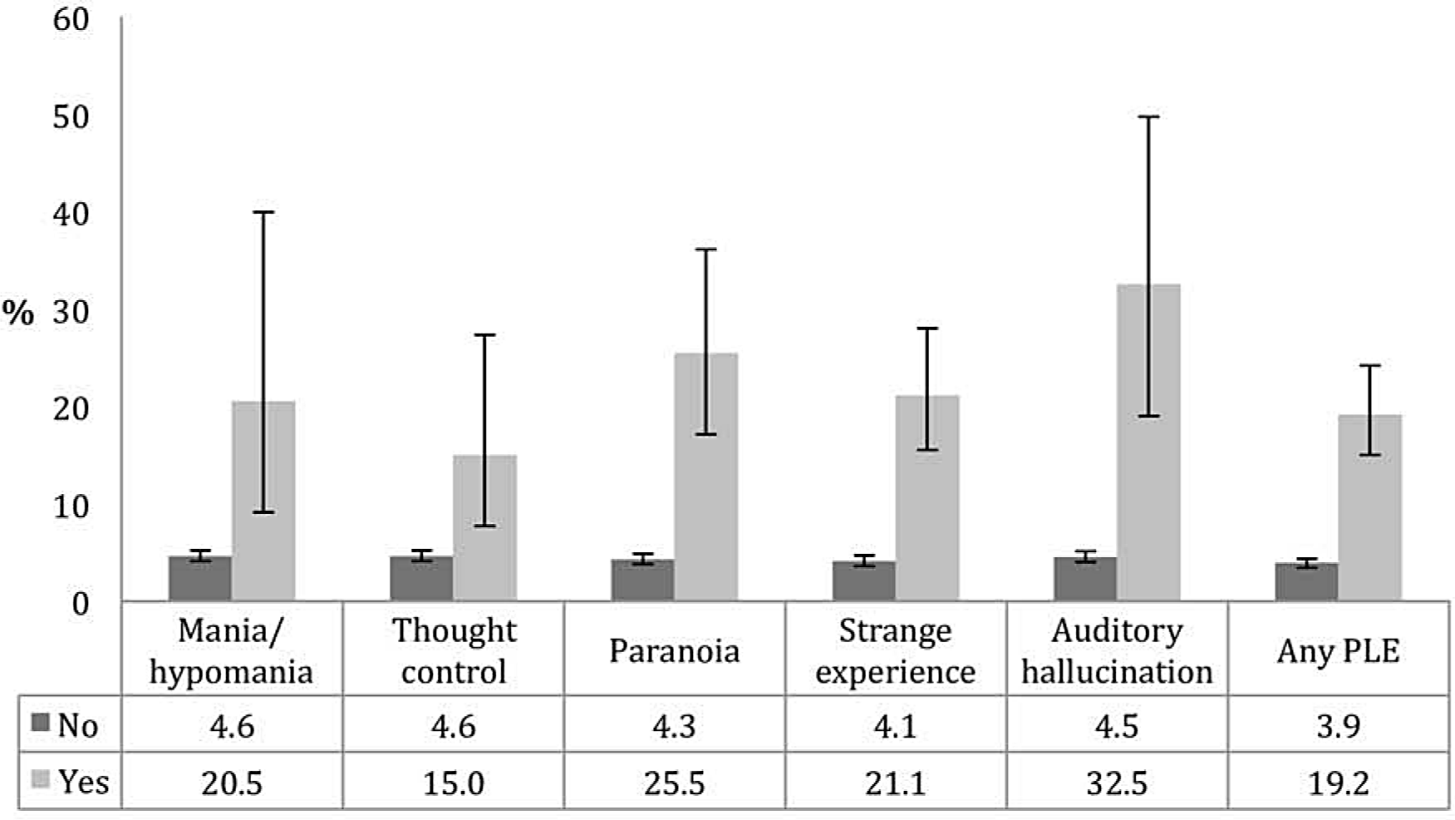
Prevalence of nonsuicidal self-injury by the presence of psychotic-like experience. Abbreviation: PLE Psychotic-like experience. Bars denote 95% confidence intervals. Prevalence figures are based on weighted sample.

The prevalence of NSSI was 4.7% (female 5.2% and male 4.2%). The characteristics of the study sample are presented in Table 1. Younger age, white British ethnic origin, lower income, alcohol and drug dependence, CMDs, a higher number of stressful life events and borderline personality disorder symptoms were associated with a significantly higher prevalence of NSSI. Fig 1 illustrates the prevalence of NSSI by the presence of PLEs. The prevalence of NSSI was significantly higher among those with any form of PLE, with prevalence figures of ≥20% observed among those with mania/hypomania (20.5%), paranoia (25.5%), strange experience (21.1%), and auditory hallucination (32.5%). The association between PLEs and NSSI estimated by multivariable logistic regression is shown in Table 2. In the model adjusted for sociodemographic factors and stressful life events (Model 1), paranoia (OR 3.57; 95%CI 1.96–6.52), thought control (OR 2.45; 95%CI 1.05–5.74), strange experience (OR 3.13; 95%CI 1.99–4.93), auditory hallucination (OR 4.03; 95%CI 1.56–10.42), and any PLE (OR 2.78; 95%CI 1.88–4.11) were significantly associated with NSSI with mania/hypomania being of borderline significance (OR 2.85; 95%CI 0.98–8.23 p = 0.054). When the models were adjusted for alcohol and drug dependence and CMDs in addition to sociodemographic factors and stressful life events (Model 2), the ORs were slightly attenuated but the associations between PLEs and NSSI remained statistically significant for paranoia, strange experience, auditory hallucination, and any PLEs with ORs ranging from 2.10 (any PLE) to 3.16 (auditory hallucination). However, the inclusion of borderline personality disorder symptoms in the model adjusted for sociodemographic factors and stressful life events largely attenuated the ORs of PLEs and rendered all previously significant ORs non-significant with the exception of paranoia (OR 2.07; 95%CI 1.08–3.96) (Model 3). The ORs of the final model adjusted for sociodemographic factors, stressful life events, alcohol and drug dependence, CMDs, and borderline personality disorder symptoms (Model 4) were similar to those of Model 3 but were largely attenuated when compared with Model 2. The results of the sensitivity analysis that used scales excluding either one or both of the criteria on suicide/self-injury and paranoia/loss of contact with reality were similar although the OR for paranoia became non-significant (OR 1.88; 95%CI 0.96–3.70) when using the scale that excluded the item on paranoia/loss of contact with reality (S2 Table).

**Table 1:** Characteristics of the study sample.

			Nonsuicidal self-injury		
Characteristic	Category	Total	No	Yes	P-value^a^
Sex	Male	48.6	48.9	43.2	0.114
	Female	51.4	51.1	56.8	
Age (years)	16–34	30.7	29.3	59.4	<0.001
	35–59	42.9	43.2	37.3	
	≥60	26.4	27.5	3.3	
Qualification^b^	No	23.8	24.0	20.2	0.161
	Yes	76.2	76.0	79.8	
Ethniticity	British White	85.2	85.0	90.9	0.020
	Other	14.8	15.0	9.1	
Income^c^	High	27.6	27.8	22.2	0.004
	Middle	25.0	25.3	19.6	
	Low	24.1	23.8	31.9	
Stressful life events^d^	Mean (SD)	3.4 (2.4)	3.3 (2.3)	5.6 (3.1)	<0.001
Alcohol dependence	No	94.2	95.0	76.9	<0.001
	Yes	5.8	5.0	23.1	
Drug dependence	No	96.6	97.2	84.7	<0.001
	Yes	3.4	2.8	15.3	
Common mental disorders^e^	No	84.0	85.6	52.6	<0.001
	Yes	16.0	14.4	47.4	
Borderline personality disorder symptoms^f^	Mean (SD)	1.2 (1.7)	1.1 (1.5)	4.1 (2.3)	<0.001

Abbreviation: SD Standard deviation

Data are % unless otherwise stated. All estimates are based on weighted data.

^a^ Differences in characteristics by the presence of nonsuicidal self-injury were tested with Chi-squared tests with the exception of stressful life events and borderline personality disorder symptoms which were tested with Student's *t*-test.

^b^ Qualification referred to degree, non-degree, A-level, GCSE, or other qualifications

^c^ Income categories were based on equivalized income tertiles (high ≥£29826, middle £14,057-<£29826, low <£14,057).

^d^ Total number of stressful life events from a list of 18 events.

^e^ Common mental disorders referred to having at least one of: depressive episode, mixed anxiety and depression, generalized anxiety disorder, panic disorder, phobia, and obsessive-compulsive disorder.

^f^ The total number of borderline personality disorder criteria endorsed. The score ranges from 0 to 9.

**Table 2:** Association between psychotic-like experience and nonsuicidal self-injury estimated by multivariable logistic regression.

	**Model 1**	**Model 2**	**Model 3**	**Model 4**		**Model 1**	**Model 2**	**Model 3**	**Model 4**
**Mania/hypomania**	2.85	2.46	1.10	1.13	**Paranoia**	3.57***	2.60**	2.07*	2.02*
	[0.98,8.23]	[0.84,7.19]	[0.34,3.52]	[0.34,3.74]		[1.96,6.52]	[1.43,4.73]	[1.08,3.96]	[1.03,3.96]
Alcohol		2.78***		1.65			2.69***		1.60
dependence		[1.79,4.30]		[0.97,2.78]			[1.74,4.18]		[0.95,2.72]
Drug dependence		1.58		1.25			1.48		1.17
		[0.94,2.66]		[0.67,2.31]			[0.88,2.51]		[0.63,2.20]
Common mental		2.43***		0.82			2.32***		0.80
disorder^a^		[1.79,3.31]		[0.56,1.21]			[1.70,3.18]		[0.54,1.17]
Borderline personality			1.65***	1.64***				1.64***	1.65***
disorder symptoms^b^			[1.55,1.76]	[1.52,1.78]				[1.54,1.76]	[1.52,1.78]
	**Model 1**	**Model 2**	**Model 3**	**Model 4**		**Model 1**	**Model 2**	**Model 3**	**Model 4**
**Thought control**	2.45*	1.99	1.23	1.19	**Strange**	3.13***	2.22***	1.21	1.27
	[1.05,5.74]	[0.80,4.99]	[0.41,3.64]	[0.39,3.63]	**experience**	[1.99,4.93]	[1.41,3.51]	[0.69,2.11]	[0.73,2.20]
Alcohol		2.69***		1.62			2.62***		1.57
dependence		[1.73,4.20]		[0.95,2.74]			[1.69,4.07]		[0.93,2.63]
Drug dependence		1.58		1.23			1.62		1.26
		[0.93,2.69]		[0.66,2.29]			[0.96,2.74]		[0.67,2.37]
Common mental		2.43***		0.81			2.15***		0.76
disorder^a^		[1.79,3.30]		[0.55,1.19]			[1.57,2.93]		[0.51,1.12]
Borderline personality			1.66***	1.65***				1.65***	1.65***
disorder symptoms^b^			[1.55,1.77]	[1.53,1.79]				[1.54,1.76]	[1.52,1.79]
	**Model 1**	**Model 2**	**Model 3**	**Model 4**		**Model 1**	**Model 2**	**Model 3**	**Model 4**
**Auditory**	4.03**	3.16*	2.07	2.23	**Any PLE**	2.78***	2.10***	1.27	1.30
**hallucination**	[1.56,10.42]	[1.21,8.21]	[0.71,5.98]	[0.75,6.65]		[1.88,4.11]	[1.42,3.10]	[0.81,1.99]	[0.83,2.04]
Alcohol		2.72***		1.63			2.67***		1.61
dependence		[1.76,4.22]		[0.96,2.76]			[1.72,4.14]		[0.96,2.72]
Drug dependence		1.58		1.24			1.54		1.23
		[0.93,2.70]		[0.66,2.32]			[0.92,2.60]		[0.66,2.28]
Common mental		2.37***		0.80			2.18***		0.79
disorder^a^		[1.76,3.20]		[0.54,1.17]			[1.60,2.96]		[0.54,1.16]
Borderline personality			1.65***	1.65***				1.64***	1.64***
disorder symptoms^b^			[1.55,1.76]	[1.52,1.79]				[1.54,1.76]	[1.52,1.78]

Abbreviation: PLE Psychotic-like experience

Data are odds ratio [95% confidence interval].

^a^ Common mental disorders referred to having at least one of: depressive episode, mixed anxiety and depression, generalized anxiety disorder, panic disorder, phobia, and obsessive-compulsive disorder.

^b^ The total number of borderline personality disorder criteria endorsed. The score ranges from 0 to 9.

Model 1: Adjusted for sex, age, education, ethnicity, income, and number of stressful life events.

Model 2: In addition to the variables in Model 1, adjusted for alcohol and drug dependence, and common mental disorders.

Model 3: In addition to the variables in Model 1, adjusted for borderline personality disorder symptoms.

Model 4: Adjusted for the variables in Model 1, alcohol and drug dependence, common mental disorders, and borderline personality disorder symptoms.

* p<0.05

** p<0.01

*** p<0.001

## Discussion

To the best of our knowledge, this is the first study on PLEs and NSSI using a large nationally-representative sample of the adult population. Approximately 5% of the population had engaged in NSSI. This prevalence is similar to previously reported figures from the U.S. general adult population [3]. Compared to those without PLEs, the prevalence of NSSI was much higher in those with PLEs. Paranoia, strange experience, auditory hallucination, and any PLEs were significantly associated with NSSI in the regression model adjusted for sociodemographic factors and stressful life events. Although alcohol and drug dependence, and CMDs had little influence on the association between PLEs and NSSI, borderline personality disorder symptoms had a strong effect as evidenced by the large attenuation of the ORs for PLEs after their inclusion in the models.

Several study limitations should be mentioned before the results are discussed. First, since all information was obtained by self-report, reporting bias may have existed. For example, social desirability may have affected the reporting of psychotic symptoms [36], and discrepancies between peer- and self-reported personality have been previously observed [37]. Second, the study would have benefited from more detailed information on PLEs and NSSI. For example, for PLEs, we did not have information on all types of PLE, their duration or the exact content of the experiences, while for NSSI we lacked information on the frequency and severity of the injury. This information would have provided more insight into the association between PLEs and NSSI. Third, the information on PLEs referred to experiences in the past 12-months whereas that relating to NSSI was on ‘lifetime ever’ experiences. Furthermore, we did not have information on potential confounding factors such as psychological distress, low self-esteem, personality traits or disorders (apart from borderline personality disorder) which are known to be associated with both PLEs and NSSI [12,38–42]. Thus, their independent and confounding effects remain unknown. Finally, as with all cross-sectional research, the temporal association or causality cannot be established.

The results of our study without adjustment for borderline personality disorder symptoms were similar to those obtained from the only community-based study on this topic to date, which took place among Australian adolescents [12] although comparability is restricted as the variables used for adjustment in the Australian study were limited, and an overall estimate for PLE regardless of the presence of psychological distress was not provided with the exception of the results on PLE persistence. In that study, in the cross-sectional analysis, after adjustment for sex and age, compared to having neither PLE nor psychological distress, the ORs (95%CIs) for PLE only, psychological distress only, and psychological distress with PLE were 2.88 (1.51–5.49), 9.77 (5.64–16.90), and 17.81 (10.00–31.71) respectively. The corresponding figures when these associations were assessed in a longitudinal fashion using data obtained one year after baseline were 1.63 (0.73–3.64), 3.22 (1.46–7.11), and 11.45 (5.70–23.00). Compared to those who had no PLEs at baseline or follow-up, those who had PLEs at both time points had an OR of 3.20 (95%CI 1.48–6.91) for incident NSSI after adjustment for sex, age, and psychological distress.

Although strong associations between mental disorders and PLEs [16,17] or NSSI [2] have been reported previously, in the current study, comorbid CMDs and substance dependence did not fully explain the association between PLEs and NSSI. Rather, borderline personality disorder symptoms had a much larger influence on this association. Thus, borderline personality disorder symptoms may be a common risk factor for both PLEs and NSSI. A high prevalence of various types of delusions and hallucinations of a transient or persistent nature have been reported in borderline personality disorder [18,19], and concurrent borderline personality features are common in people at ultra high risk for psychosis [19], while NSSI has been associated with borderline personality disorder or borderline personality disorder symptoms [4] even when the diagnostic criterion of suicide/self-injury is excluded [20]. Borderline personality disorder symptoms include features such as unstable and intense relationships, impulsive and self-destructive behaviors, anger-related problems, and affective instability [8] and these characteristics may explain the strong influence of borderline personality disorder symptoms in the association between PLEs and NSSI. For example, it has been suggested that interpersonal functioning may play a pivotal role in the emergence of both delusions and hallucinations in borderline personality disorder where anger and hostility against others may underlie paranoid delusions, and avoidance of interpersonal relationships may lead to hallucinations [18], while interpersonal difficulties have been associated with NSSI [43]. Furthermore, stress-induced psychotic reactivity is common in borderline personality disorder [44] and stress is a risk factor for NSSI [45]. Finally, impulsivity and emotion dysregulation may underlie both PLEs and NSSI [19,46–48].

In conclusion, this study showed that borderline personality disorder symptoms may be an important factor in the link between PLEs and NSSI. Future studies assessing the association between PLEs and NSSI may benefit from taking borderline personality disorder symptoms into account. Studies with a longitudinal design are warranted to determine the temporal relationship and causality, and will be essential to understand how borderline personality disorder symptoms, PLEs, and NSSI are inter-related.

## Supporting Information

S1 Table Stressful life events. (DOCX)

S2 Table Sensitivity analyses of the association between psychotic-like experiences and nonsuicidal self-injury. (DOCX)
