# Correction: Psychotic-Like Experiences and Nonsuidical Self-Injury in England: Results from a National Survey

**DOI:** 10.1371/journal.pone.0147095

**Published:** 2016-01-11

**Authors:** Ai Koyanagi, Andrew Stickley, Josep Maria Haro

The word “Nonsuicidal” is misspelled in the title. The correct title is: Psychotic-Like Experiences and Nonsuicidal Self-Injury in England: Results from a National Survey. The correct citation is: Koyanagi A, Stickley A, Haro JM (2015) Psychotic-Like Experiences and Nonsuicidal Self-Injury in England: Results from a National Survey. PLoS ONE 10(12): e0145533. doi:10.1371/journal.pone.0145533
