# Supplementary material for: Psychotic-Like Experiences and Nonsuidical Self-Injury in England: Results from a National Survey
Source: PLoS One. 2015 Dec 23;10(12):e0145533. doi: 10.1371/journal.pone.0145533 (PMC4689421; doi:10.1371/journal.pone.0145533)
Supplement: S1 Table — (DOCX) [file pone.0145533.s001.docx]

| **S1 Table. Stressful life events.** | |
| --- | --- |
| 1 | Serious illness, injury or assault to yourself |
| 2 | Serious illness, injury or assault to a close relative |
| 3 | Death of an immediate family member of yours |
| 4 | Death of a close family friend or other relative, like an Aunt, cousin or grandparent |
| 5 | Separation due to marital difficulties, divorce or steady relationship broken down |
| 6 | Serious problem with a close friend, neighbor or relative |
| 7 | Being made redundant or sacked from your job |
| 8 | Looking for work without success for more than 1 month |
| 9 | Major financial crisis, like losing the equivalent of 3 months income |
| 10 | Problem with police involving court appearance |
| 11 | Something you valued being lost or stolen |
| 12 | Bullying |
| 13 | Violence in the home |
| 14 | Violence at work |
| 15 | Sexual abuse |
| 16 | Being expelled from school |
| 17 | Running away from your home |
| 18 | Being homeless |
