# Supplementary material for: Psychotic-Like Experiences and Nonsuidical Self-Injury in England: Results from a National Survey
Source: PLoS One. 2015 Dec 23;10(12):e0145533. doi: 10.1371/journal.pone.0145533 (PMC4689421; doi:10.1371/journal.pone.0145533)
Supplement: S2 Table — (DOCX) [file pone.0145533.s002.docx]

**S2 Table. Sensitivity analyses of the association between psychotic-like experiences and nonsuicidal self-injury.**

| Association between psychotic-like experiences and nonsuicidal self-injury estimated by multivariable logistic regression using the borderline personality disorder symptom scale without suicide/self-injury | | | | | |
| --- | --- | --- | --- | --- | --- |
| Mania/hypomania | 1.24 | Paranoia | 2.33* | Auditory | 2.61 |
|  | [0.36,4.24] |  | [1.21,4.48] | hallucination | [0.88,7.76] |
| Alcohol dependence | 1.92* |  | 1.86* |  | 1.89* |
|  | [1.16,3.18] |  | [1.12,3.09] |  | [1.14,3.14] |
| Drug dependence | 1.32 |  | 1.23 |  | 1.31 |
|  | [0.72,2.39] |  | [0.67,2.25] |  | [0.71,2.41] |
| Common mental | 1.08 |  | 1.04 |  | 1.04 |
| disorders^a^ | [0.74,1.58] |  | [0.71,1.53] |  | [0.71,1.53] |
| Borderline Borderline personality disorder symptoms^b^ | 1.46*** |  | 1.46*** |  | 1.47*** |
|  | [1.35,1.59] |  | [1.34,1.60] |  | [1.35,1.60] |
|  |  |  |  |  |  |
| Thought control | 1.32 | Strange | 1.52 | Any PLE | 1.49 |
|  | [0.44,3.99] | experience | [0.88,2.62] |  | [0.96,2.32] |
| Alcohol dependence | 1.87* |  | 1.82* |  | 1.87* |
|  | [1.13,3.11] |  | [1.11,2.99] |  | [1.13,3.09] |
| Drug dependence | 1.30 |  | 1.35 |  | 1.30 |
|  | [0.71,2.38] |  | [0.73,2.48] |  | [0.71,2.35] |
| Common mental | 1.06 |  | 0.98 |  | 1.03 |
| disorders^a^ | [0.73,1.56] |  | [0.67,1.44] |  | [0.70,1.50] |
| Borderline personality disorder symptoms^b^ | 1.47*** |  | 1.47*** |  | 1.46*** |
|  | [1.35,1.60] |  | [1.35,1.60] |  | [1.34,1.59] |

Abbreviation: PLE Psychotic-like experience

Data are odds ratio [95% confidence interval].

^a^ Common mental disorders referred to having at least one of: depressive episode, mixed anxiety and depression, generalized anxiety disorder, panic disorder, phobia, and obsessive-compulsive disorder.

^b^ The total number of borderline personality disorder criteria endorsed (Cronbach's alpha 0.74). The score ranges from 0 to 8.

Models are adjusted for sex, age, education, ethnicity, income, and number of stressful life events, alcohol and drug dependence, common mental disorders, and borderline personality disorder symptoms.

* p<0.05, *** p<0.001

| Association between psychotic-like experiences and nonsuicidal self-injury estimated by multivariable logistic regression using the borderline personality disorder symptom scale without paranoia/loss of contact with reality | | | | | |
| --- | --- | --- | --- | --- | --- |
| Mania/hypomania | 1.03 | Paranoia | 1.88 | Auditory | 2.31 |
|  | [0.33,3.26] |  | [0.96,3.70] | hallucination | [0.81,6.60] |
| Alcohol dependence | 1.73* |  | 1.70* |  | 1.71* |
|  | [1.04,2.89] |  | [1.02,2.83] |  | [1.03,2.86] |
| Drug dependence | 1.26 |  | 1.19 |  | 1.25 |
|  | [0.68,2.32] |  | [0.64,2.22] |  | [0.67,2.33] |
| Common mental | 0.86 |  | 0.83 |  | 0.83 |
| disorders^a^ | [0.59,1.25] |  | [0.57,1.21] |  | [0.57,1.21] |
| Borderline personality disorder symptoms^b^ | 1.72*** |  | 1.72*** |  | 1.72*** |
|  | [1.58,1.87] |  | [1.58,1.88] |  | [1.58,1.88] |
|  |  |  |  |  |  |
| Thought control | 1.37 | Strange | 1.27 | Any PLE | 1.31 |
|  | [0.54,3.50] | experience | [0.74,2.17] |  | [0.85,2.03] |
| Alcohol dependence | 1.69* |  | 1.66* |  | 1.69* |
|  | [1.01,2.84] |  | [1.00,2.74] |  | [1.02,2.82] |
| Drug dependence | 1.24 |  | 1.27 |  | 1.24 |
|  | [0.67,2.30] |  | [0.68,2.39] |  | [0.67,2.29] |
| Common mental | 0.84 |  | 0.79 |  | 0.82 |
| disorders^a^ | [0.58,1.23] |  | [0.54,1.16] |  | [0.56,1.20] |
| Borderline personality disorder symptoms^b^ | 1.73*** |  | 1.73*** |  | 1.72*** |
|  | [1.59,1.88] |  | [1.58,1.88] |  | [1.58,1.87] |

Abbreviation: PLE Psychotic-like experience

Data are odds ratio [95% confidence interval].

^a^ Common mental disorders referred to having at least one of: depressive episode, mixed anxiety and depression, generalized anxiety disorder, panic disorder, phobia, and obsessive-compulsive disorder.

^b^ The total number of borderline personality disorder criteria endorsed (Cronbach's alpha 0.72). The score ranges from 0 to 8.

Models are adjusted for sex, age, education, ethnicity, income, and number of stressful life events, alcohol and drug dependence, common mental disorders, and borderline personality disorder symptoms.

* p<0.05, *** p<0.001

| Association between psychotic-like experiences and nonsuicidal self-injury estimated by multivariable logistic regression using the borderline personality disorder symptom scale without suicide/self-injury and paranoia/loss of contact with reality | | | | | |
| --- | --- | --- | --- | --- | --- |
| Mania/hypomania | 1.18 | Paranoia | 2.20* | Auditory | 2.71 |
|  | [0.36,3.86] |  | [1.14,4.22] | hallucination | [0.94,7.85] |
| Alcohol dependence | 2.04** |  | 1.99** |  | 2.01** |
|  | [1.25,3.34] |  | [1.21,3.26] |  | [1.23,3.30] |
| Drug dependence | 1.34 |  | 1.25 |  | 1.33 |
|  | [0.74,2.42] |  | [0.69,2.28] |  | [0.73,2.44] |
| Common mental | 1.14 |  | 1.09 |  | 1.09 |
| disorders^a^ | [0.79,1.66] |  | [0.75,1.59] |  | [0.75,1.59] |
| Borderline personality disorder symptoms^b^ | 1.49*** |  | 1.49*** |  | 1.50*** |
|  | [1.36,1.64] |  | [1.36,1.64] |  | [1.37,1.65] |
|  |  |  |  |  |  |
| Thought control | 1.56 | Strange | 1.55 | Any PLE | 1.52 |
|  | [0.61,4.00] | experience | [0.91,2.63] |  | [0.99,2.34] |
| Alcohol dependence | 1.98** |  | 1.95** |  | 1.99** |
|  | [1.20,3.27] |  | [1.20,3.16] |  | [1.22,3.25] |
| Drug dependence | 1.32 |  | 1.37 |  | 1.32 |
|  | [0.73,2.40] |  | [0.75,2.50] |  | [0.73,2.37] |
| Common mental | 1.12 |  | 1.03 |  | 1.07 |
| disorders^a^ | [0.77,1.63] |  | [0.70,1.50] |  | [0.74,1.56] |
| Borderline personality disorder symptoms^b^ | 1.50*** |  | 1.50*** |  | 1.49*** |
|  | [1.37,1.65] |  | [1.36,1.65] |  | [1.35,1.63] |

Abbreviation: PLE Psychotic-like experience

Data are odds ratio [95% confidence interval].

^a^ Common mental disorders referred to having at least one of: depressive episode, mixed anxiety and depression, generalized anxiety disorder, panic disorder, phobia, and obsessive-compulsive disorder.

^b^ The total number of borderline personality disorder criteria endorsed (Cronbach's alpha 0.72). The score ranges from 0 to 7.

Models are adjusted for sex, age, education, ethnicity, income, and number of stressful life events, alcohol and drug dependence, common mental disorders, and borderline personality disorder symptoms.

* p<0.05, ** p<0.01, *** p<0.001
